# Crucial involvement of fast waves and Delta band in the brain network attributes of infantile epileptic spasms syndrome

**DOI:** 10.3389/fped.2023.1249789

**Published:** 2023-10-20

**Authors:** Yan Dong, Liang Jin, Mengchun Li, Ruofei Lian, Gongao Wu, Ruijuan Xu, Xiaoli Zhang, Kaixian Du, Tianming Jia, Haiyan Wang, Shichao Zhao

**Affiliations:** ^1^ Department of Pediatrics, The Third Affiliated Hospital of Zheng Zhou University, Zhengzhou, China; ^2^ Henan Key Laboratory of Child Brain Injury and Henan Pediatric Clinical Research Center, Third Affiliated Hospital and Institute of Neuroscience of Zhengzhou University, Zhengzhou, China; ^3^ Department of Pediatrics, Zhumadian Central Hospital, Zhumadian, China

**Keywords:** fast waves, Delta band, analysis, functional network, graph theory, infantile epileptic spasms syndrome

## Abstract

**Objective:**

This study aims to describe the characteristics of the brain network attributes in children diagnosed with Infantile Epileptic Spasms Syndrome (IESS) and to determine the influence exerted by adrenocorticotrophic hormone (ACTH) or methylprednisolone (MP) on network attributes.

**Methods:**

In this retrospective cohort study, we recruited 19 infants diagnosed with IESS and 10 healthy subjects as the control from the Pediatric Neurology Department at the Third Affiliated Hospital of Zhengzhou University between October 2019 and December 2020. The first thirty-minute processed electroencephalograms (EEGs) were clipped and filtered into EEG frequency bands (2 s each). A comparative assessment was conducted between the IESS group and the controls as well as the pre- and post-treatment in the IESS group. Mutual information values for each EEG channel were collected and compared including characteristic path length (CPL), node degree (ND), clustering coefficient (CC), and betweenness centrality (BC), based on graph theory.

**Results:**

Comparing the control group, in the IESS group, there was an increase in CPL of the Delta band, and a decrease in ND and CC of the Delta band during the waking period, contrary to those during the sleeping period (*P* < 0.05), a decreased in CPL of the fast waves and an increase in ND and CC (*P* < 0.05) in the sleep-wake cycle, and a decrease in ND and CC of the Theta band in the waking phase. Post-treatment compared with the pre-treatment, during the waking ictal phase, there was a noted decrease in CPL in the Delta band and fast waves, while an increase was observed in ND and CC (*P* < 0.05).

**Conclusions:**

The Delta band and fast waves are crucial components of the network attributes in IESS.

**Significance:**

This investigation provides a precise characterization of the brain network in children afflicted with IESS, and lays the groundwork for predicting the prognosis using graph theory.

## 1. Introduction

Infantile Epileptic Spasm Syndrome (IESS), otherwise known as infantile spasms (IS), presents as a severe form of epilepsy, characterized by developmental lags and recurrent seizures (1). The syndrome is typified by epileptic spasms, arrested development or regression, and hypsarrhythmia observable in interictal electroencephalogram (EEG) recordings (2). There is potential for IESS to be associated with visual biomarkers such as the evaluation of hypsarrhythmia, epileptiform discharges (EDs), and the Burden of Amplitudes and Epileptiform Discharges (BASED) (3–5). It has been well-established that the overall EEG complexity in IESS patients is inferior to that of healthy controls, whereas the full EEG complexity in patients responding to Adrenocorticotrophic Hormone (ACTH) therapy surpasses that of non-responders (6). The use of scalp high-frequency oscillations (HFOs) emerges as a potent biomarker for assessing the effectiveness and prognostic potential of ACTH therapy in IESS patients (7). Given the challenge of managing seizures and the elevated rate of recurrence, prediction of treatment response or IESS relapse has become of paramount importance. Though a few studies have been reported, the available studies have not reported the biomarkers in detail or shown how ACTH or the alternative methylprednisolone (MP) affects brain network attributes. Therefore, it has significant implications to analyze Brain Functional Network (BFN) attributes of IESS, both prior to and following hormonal therapy.

BFN stands as a pivotal approach in the treatment of epilepsy, extensively employed to probe functional alterations within the brain (8–10). Previous investigations have identified a correlation between tuber locations and bilateral globi pallidi associated with IESS (3). Epileptic scalp fast oscillations have been validated as indicators of the severity of epileptic encephalopathy, especially with regard to IESS (11). High-frequency scalp oscillations have proven to be efficacious biomarkers for gauging the effectiveness and prognostic capacity of ACTH therapy in IESS patients (7). The transmission capacity of fast waves, defensive prowess, and local connectivity of slow waves all manifested an enhancement during IESS ictal onset (12). Nevertheless, the exploration of BFN alterations in children diagnosed with IESS, contrasted with healthy infants, remains inadequate. The implications of hormonal treatment on the disrupted BFN, inclusive of interictal and ictal EEG modifications, remain nebulous. As such, there exists an exigent need for further study, aimed at a comprehensive analysis of network attributes in children suffering from IESS pre- and post-ACTH or MP pulse treatment to leverage graph theory.

This study aims to provide a fresh perspective to comprehend the idiosyncrasies of IESS by analyzing the transformations of brain networks in children with IESS and healthy controls, as well as IESS patients between pre- and post-ACTH or MP treatment. This could potentially yield invaluable insights for future clinical intervention and timely prognostic evaluation.

## 2. Materials and methods

### 2.1. Participants

This study enrolled 19 hospitalized participants (eight males and eleven females), all diagnosed with IESS and 10 healthy controls from the Pediatric Neurology Department at the Third Affiliated Hospital of Zhengzhou University, between October 2019 and December 2020. IESS diagnosis was made in accordance with the International League Against Epilepsy (ILAE) guidelines. The inclusion criteria entailed: (1) display of epileptic spasms as the sole seizure type during infancy; (2) interictal EEG indicative of hypsarrhythmia; (3) significant developmental delay or regression; (4) availability of an EEG recorded before and after the initial treatment with ACTH or MP; (5) a follow-up period exceeding 12 months after hormonal therapy; and (6) comprehensive clinical data. The exclusion criteria included: (1) concurrent severe illnesses; (2) termination of treatment; (3) loss to follow-up or withdrawal from clinical observation, as per parental request. Ten control subjects with normal EEG were comparable in age and sex to the IESS patient. The study was approved by the Research Ethics Committees at the Third Affiliated Hospital of Zhengzhou University, Zhengzhou, China (reference: 2021-042-01). Written informed consent from the legal guardian or next of kin was waived by the committee, given the retrospective nature of the study and the minimal risk to participants.

The enrolled children with IESS were followed up until July 1, 2022. A structured clinical information form was utilized to gather the demographic and medical history data, including the age at the first onset of IESS, the administration of ACTH/MP and anti-seizure medications (ASMs), and results of video electroencephalogram (VEEG), cranial magnetic resonance imaging (MRI). The results of Griffiths Mental Development Scales-Chinese (GDS-C) were also collected which measures the rate of development of motor, personal-social, language, eye-hand coordination, performance and practical reasoning for children from birth to eight years of age, and any requisite genetic testing.

### 2.2. BASED scores of EEG

Scalp 32-Channel VEEG (16 h/24 h) data were collected using electrodes placed according to the international 10/20 system, sampled at 500 Hz (Nihon-Kohden, Tokyo, Japan). EEG data was examined by two clinical neurophysiologists certified by the China Association Against Epilepsy. Discrepancies were resolved by a third one. The 2021 BASED score system was employed to compute pretreatment and posttreatment scores (13) using a scale of 0–5, with 0 for normal activity and 5 for the presence of >3 spike foci occupying >50% of one-second bins.

### 2.3. EEG data acquisition and preprocessing

Ictal and interictal EEG clips lasting the first 30 min from the processed EEGs of different phases, encompassing isolated or clustered epileptic spasms, were filtered into EEG frequency bands (2s each)—Delta (0.3–3.5 Hz), Theta (4–7.5 Hz), Alpha (8–13 Hz), Beta (14–30 Hz), and Gamma (30–70 Hz). These selected EEGs were then divided into 900 epochs (1 epoch per 2 s). Mutual information values for each channel of EEG, such as characteristic path length (CPL), node degree (ND), clustering coefficient (CC), and betweenness centrality (BC), were directly compared between the IESS patients and controls, as well as before and after ACTH or MP treatment, as shown in Supplementary Table S1.

Detailed introduction to the BFN of patients with IESS, based on graph theory combined with Weighted Phase Lag Index (wPLI), can be found in our previous study (12) and Supplementary Table S1. CPL represents the average path length of all nodes in the network, indicating the information transmission capability of the whole-brain network. A shorter CPL implies a stronger information transfer capacity. ND reflects the local connectivity of a node in the network. CC measures the capacity for local information transmission in the network and indicates the network's resilience against random attacks. BC defines node centrality in terms of information flow and reflects a node's hub status in the local network.

For the raw EEG data, an average reference was first applied to the band-pass filter (0.5–45 Hz) using the EEGLAB toolkit (https://sccn.ucsd.edu/eeglab/index.php). The filtered signal was then subjected to independent component analysis. Artifact data were removed using the SASICA toolkit (https://eeglab.org/others/EEGLAB_Extensions.html) to obtain the pre-processed EEG signal. Finally, the BCT toolkit (https://www.nitrc.org/projects/bct) was used to determine CPL, ND, CC and BC.

### 2.4. Group statistical comparisons

The pairwise comparative EEGs assessment of the waking/sleep phase between the IESS group and the control; the interical/icatal phase of IESS group between pre-treatment and post-treatment, was conducted. EEG data analyses were performed using MATLAB software (the MathWorks Inc., Natick, MA, USA), and network attributes were compared using a two-sided non-parametric Wilcoxon rank sum test.

Demographic data and BASED scores were analyzed using SPSS 26.0 software (SPSS Inc., Chicago, IL, USA). Count data were presented as proportions and analyzed via the chi-square test. Measured clinical data were displayed as mean ± standard deviation, with Student's *t*-test employed for normally distributed data. Median (interquartile range) values were reported for non-normally distributed data, analyzed via Wilcoxon's rank-sum test. *P* < 0.05 was considered statistically significant.

## 3. Results

### 3.1. Clinical and demographic data

The average age at enrollment for the IESS group was 6.93 (5.77–10.77) months, while that was 7.00 ± 3.36 months in the control group. There was no significant difference in age or sex between the two groups (*P* > 0.05). All patients in the IESS group exhibited developmental delays, with GDS-C *Z*-scores below −2.

**Figure 1:**
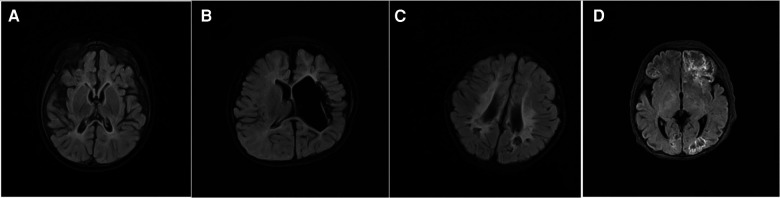
Some typical magnetic resonance imaging (MRI) findings in the patients with infantile epileptics spasms syndrome (IESS), T2 weighted imaging (T2 WI flair), (**A**) MRI (8-month-old) showed bilateral occipital encephalomalacia, brain atrophy, hyperintensity in the external capsule. (**B**) MRI (17-month-old) showed bilateral periventricular leukomalacia. (**C**) MRI (7-month-old) showed left hemisphere parenchyma, and left ventricular dilatation. (**D**) MRI (10-day-old) exhibited fronto-temporo-parietal-occipital encephalomalacia.

In the IESS group, structural etiology was the most prevalent, accounting for 47.37% (9 cases), among which 77.78% (7 cases) were due to encephalomalacia (five resulting from birth hypoxia, and two from postnatal hypoglycemia) (Figure 1). Six cases (31.58%) had an unknown etiology, and four cases (21.05%) had genetic etiology (one each with methylmalonic acidemia, Down syndrome, maternal heterozygous GABRB3 variation, and *de novo* heterozygous IRF2BP variation). There were no instances of infectious or immunological etiology.

In the IESS group, fourteen patients received MP pulse treatment, while the remaining five were treated with ACTH pulse therapy. In our cohort, the long-term control rate of IESS with structural etiology was lower than that of IESS with an unknown etiology, although the difference was not statistically significant (*P* > 0.05). Seventeen patients (89.47%) showed varying degrees of developmental delay.

### 3.2. Video electroencephalogram and BASED scores

Interictal phase VEEG displayed hypsarrhythmia, with spasm attacks observed in the IESS children. The mean BASED score was 4.83 ± 0.38 before hormonal therapy and 3.44 ± 0.82 after therapy, with a statistically significant reduction (*P* < 0.01). After more than a year of post-treatment follow-up, no spasms were detected in six cases, eight cases reported a decreased frequency of spasms, and five cases showed no significant reduction in spasms.

### 3.3. Brain functional network of IESS

#### 3.3.1. The comparisons between the IESS and control group

##### 3.3.1.1. During the waking phase

**Figure 2:**
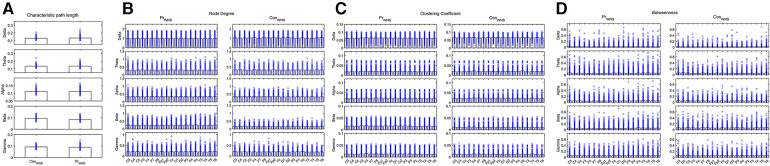
The brain network attributes of IESS and control groups during wakefulness. (**A**): Characteristic path length (CPL). (**B**): Node degree (ND). (**C**): Clustering coefficient (CC). (**D**): Betweenness centrality (BC). PrWNS: network attributes of IESS group during wakefulness, ConWNS: network attributes of control group during wakefulness.

Compared the brain network properties with the control group, the CPL of the Delta band increased, and the CPL of the Beta and Gamma bands decreased (*P* < 0.05) in the IESS group. The ND and CC of the Delta and Theta bands were decreased, though they were increased in the fast wave band (*P* < 0.05) in the IESS group. The BC in the temporal region of the Delta band increased but decreased in the fast wave band (*P* < 0.05) in the IESS group, as shown in Figure 2 and Table 1.

**Table 1:** Comparison of brain network attributes properties between IESS and control groups during wakefulness.

Band	CPL	ND	CC	BC
Delta	↑*	FP2↓*, F3↓*, F4↓*, C3↓*, P3↓*, O1↓*, O2↓*, T5↓*, T6↓*, Pz↓*	FP2↓*, F3↓*, C3↓*, P3↓*, O1↓*, T3↓*, T5↓*, Fz↓*, Pz↓*	P4↑*, T3↑*, T4↑*, T6↑*
Theta	–	C3↓*, C4↓*, P3↓*	T5↓*, Pz↓*	C4↓*, O1↑*, F7↓*
Alpha	–	O1↑*, T5↑*	–	F4↓*, O1↑*, T3↑*, T5↑*
Beta	↓*	FP1↑*, FP2↑*, F3↑*, F4↑*, C3↑*, C4↑*, P3↑*, P4↑*, O1↑*, O2↑*, F7↑*, F8↑*, T3↑*, T4↑*, T5↑*, T6↑*, Fz↑*, Cz↑*, Pz↑*	FP1↑*, FP2↑*, F3↑*, F4↑*, C3↑*, C4↑*, P3↑*, P4↑*, O1↑*, O2↑*, F7↑*, F8↑*, T3↑*, T4↑*, T5↑*, T6↑*, Fz↑*, Cz↑*, Pz↑*	FP1↓*, FP2↓*, F3↓*, F4↓*, P3↓*, F7↓*, T6↓*, Cz↓*
Gamma	↓*	O2↑*	FP1↑*, F3↑*, P3↑*, F8↑*, Fz↑*	F3↓*

Two-sided nonparametric Wilcoxon rank-sum tests were taken. The local connectivity and defense ability of the fast waves were enhanced in children with IESS. The overall transmission efficiency, local connectivity, and defense capacity of the Delta band decreased. ↑ represents increasing or expanding, and ↓ represents decreasing or shortening. BC, betweenness centrality; CC, clustering coefficient; CPL, characteristic path length.

* Indicates statistical significance at *P *< 0.05.

##### 3.3.1.2. During the sleep phase

**Figure 3:**
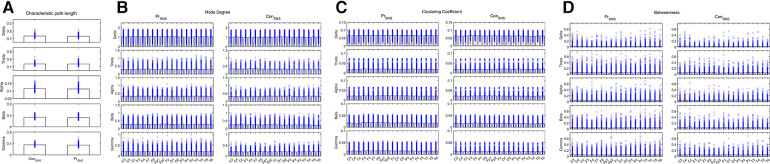
Network attributes of IESS and control groups during the sleep phase. (**A**): CPL. (**B**): ND (**C**): CC. (**D**): BC. PrSNS: network attributes of IESS group during the sleep phase, ConSNS: network attributes of control group during the sleep phase.

The CPL of the Delta and Beta bands decreased in the IESS group compared with the control group. The ND and CC of the Delta, Alpha, Beta and Gamma bands were increased while decreased in the Theta band, and the BC decreased in the frontal area (All *P* < 0.05) in the IESS group (Figure 3, Table 2).

**Table 2:** Comparison of network attributes between IESS and control groups during the sleep phase.

Band	CPL	ND	CC	BC
Delta	↓*	FP2↑*, F3↑*, F4↑*, C3↑*, P4↑*, F7↑*, F8↑*, T3↑*, T4↑*, Fz↑*, Cz↑*	FP1↑*, FP2↑*, F3↑*, F4↑*, C3↑*, C4↑*, P3↑*, O1↑*, O2↑*, F7↑*, T3↑*, T4↑*, T5↑*, T6↑*, Cz↑*, Pz↑*	FP1↓*, P3↓*, P4↓*, Pz↓*
Theta	–	F4↓*, C3↓*, C4↓*, O2↓*, F7↓*, T4↓*	O1↓*	FP1↓*, C3↓*
Alpha	–	F3↑*, T3↑*	F3↑*	FP1↓*, P3↓*, O1↓*
Beta	↓*	FP1↑*, FP2↑*, F3↑*, F4↑*, C3↑*, P3↑*, P4↑*, O1↑*, O2↑*, F7↑*, F8↑*, T3↑*, T4↑*, T5↑*, T6↑*, Pz↑*	FP1↑*, FP2↑*, F3↑*, F4↑*, C3↑*, C4↑*, P3↑*, P4↑*, O2↑*, F7↑*, F8↑*, T3↑*, T4↑*, T5↑*, T6↑*, Cz↑*, Pz↑*	F4↓*, C4↓*, O1↓*, T6↑*, Fz↓*, Cz↓*
Gamma	–	FP1↑*, FP2↑*, F3↑*, C3↑*, C4↑*, P3↑*, P4↑*, Pz↑*	FP1↑*, FP2↑*, F3↑*, F4↑*, C4↑*, P3↑*, P4↑*, O1↑*, F7↑*, F8↑*, T6↑*, Fz↑*, Cz↑*, Pz↑*	F4↓*, T4↓*

Two-sided nonparametric Wilcoxon rank-sum tests were taken. The local connectivity and defense ability of the fast waves were enhanced.

* Indicates statistical significance at *P *< 0.05.

#### 3.3.2. The comparison between before and after hormonal therapy in the IESS group

##### 3.3.2.1. During wakefulness

**Figure 4:**
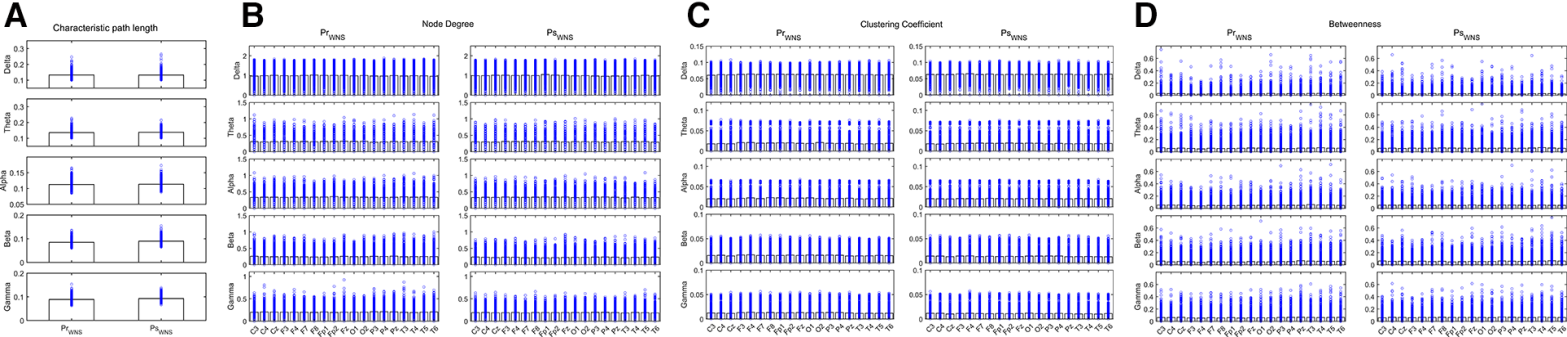
Network attributes in the IESS group during wakefulness before and after hormonal therapy. (**A**): CPL. (**B**): ND (**C**): CC. (**D**): BC. PrWNS: network attributes of IESS group during wakefulness before hormonal therapy. PsWNS: network attributes of the control group during wakefulness after hormonal treatment.

During wakefulness when compared the brain network properties of the IESS group before hormonal therapy with that after hormonal therapy, the CPL for the Beta and Gamma bands increased after therapy. The ND and CC for the Theta, Alpha, Beta, and Gamma bands decreased after therapy, and the BC for the Delta, Theta, Beta, and Gamma bands increased after therapy after hormonal therapy. However, the BC for the temporal region of the Alpha band decreased (all *P* < 0.05) (as shown in Figure 4 and Table 3).

**Table 3:** Comparison of network attributes before and after hormonal therapy during wakefulness in the IESS group.

Band	CPL	ND	CC	BC
Delta	–	FP1↑*	–	FP1↑*, FP2↑*, F4↑*, F7↑*, Cz↑*
Theta	–	Fz↓*	F3↓*, F4↓*, O2↓*	F4↑*, C4↑*, P3↑*, T5↑*
Alpha	–	FP2↓*, P4↓*, O1↓*, T3↓*, T5↓*	FP1↓*, FP2↓*, F4↓*, O1↓*, F8↓*, T5↓*, Pz↓*	FP1↑*, F3↑*, F4↑*, P4↑*, F8↑*, T3↓*, T5↓*, Fz↑*, Pz↑*
Beta	↑*	FP1↓*, FP2↓*, C3↓*, C4↓*, P3↓*, P4↓*, O1↓*, O2↓*, F7↓*, F8↓*, T3↓*, T4↓*, T5↓*, Fz↓*, Cz↓*, Pz↓*	FP1↓*, FP2↓*, F3↓*, F4↓*, C3↓*, C4↓*, P3↓*, P4↓*, O1↓*, O2↓*, F7↓*, F8↓*, T3↓*, T4↓*, T5↓*, T6↓*, Fz↓*, Cz↓*, Pz↓*	FP2↑*, F3↑*, F4↑*, C4↑*, P4↑*, O1↑*, F8↑*, T6↑*
Gamma	↑*	FP1↓*, F3↓*, P3↓*, F7↓*, T3↓*, T5↓*, T6↓*, Cz↓*	FP1↓*, FP2↓*, F3↓*, F4↓*, C4↓*, P3↓*, P4↓*, O1↓*, O2↓*, F7↓*, F8↓*, T3↓*, T4↓*, T5↓*, T6↓*, Fz↓*, Cz↓*, Pz↓*	FP2↑*, F4↑*, F8↑*, Fz↑*

Two-sided nonparametric Wilcoxon rank-sum tests were taken. MP and ACTH treat the brain network dysfunction by reducing the overall transmission efficiency, local connectivity and defense ability of the fast waves in IESS children, and reducing the local connectivity and defense ability of the Theta band.

* Indicates statistical significance at *P *< 0.05.

##### 3.3.2.2. During the sleep phase

**Figure 5:**
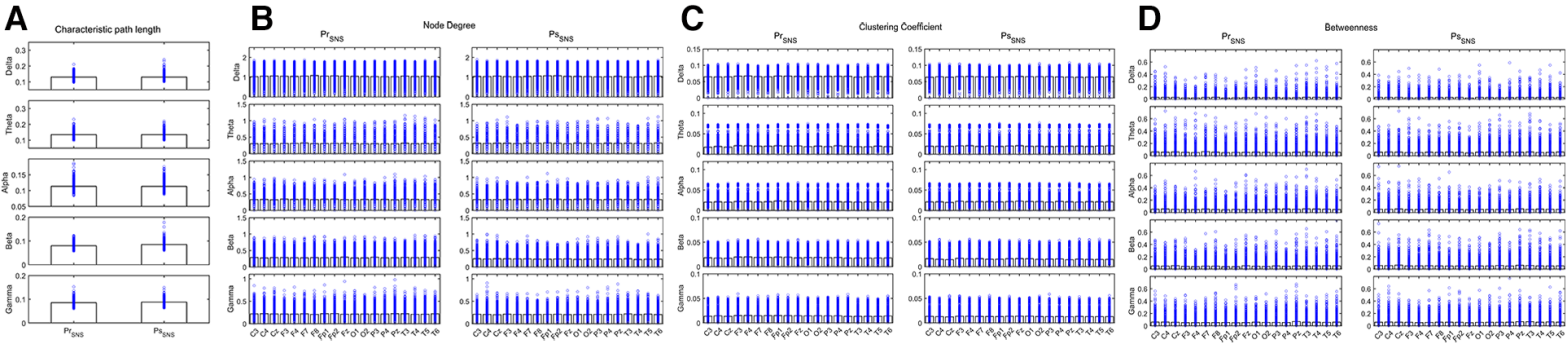
Network attributes in IESS group during the sleep phase before and after hormonal therapy. (**A**): CPL. (**B**): ND (**C**): CC. (**D**): BC. PrSNS: network attributes of IESS group during the sleep phase before hormonal therapy. PsSNS: network attributes of the control group during the sleep phase after hormonal therapy.

Compared the IESS group before hormonal therapy, the CPL of the Beta band increased, the ND and CC decreased in the Delta, Beta, and Gamma bands, and the BC increased in the Beta and Gamma bands in the IESS group during the sleep phase following ACTH or MP pulse therapy (all *P* < 0.05) (as shown in Figure 5 and Table 4).

**Table 4:** Comparison of network attributes before and after hormonal therapy during the sleep phase.

Band	CPL	ND	CC	BC
Delta	–	T3↓*	FP1↓*, FP2↓*, P4↓*, Pz↓*	FP1↑*, FP2↑*, Fz↓*, Pz↑*
Theta	–	FP1↑*, P4↑*, T6↑*, Cz↑*	C3↑*, T6↑*, Cz↑*	FP1↑*, P4↑*, Fz↓*
Alpha	–	Pz↑*	FP1↓*	–
Beta	↑*	FP1↓*, FP2↓*, F3↓*, F4↓*, C3↓*, C4↓*, P3↓*, P4↓*, O1↓*, O2↓*, F7↓*, F8↓*, T3↓*, T4↓*, T5↓*, T6↓*, Fz↓*, Cz↓*, Pz↓*	FP1↓*, FP2↓*, F3↓*, F4↓*, C3↓*, C4↓*, P3↓*, P4↓*, O1↓*, O2↓*, F7↓*, F8↓*, T3↓*, T4↓*, T5↓*, T6↓*, Fz↓*, Cz↓*, Pz↓*	FP1↑*, FP2↑*, F3↑*, F4↑*, C4↑*, O2↑*, T3↑*, Pz↑*
Gamma	–	FP1↓*, C4↓*, T6↓*, Cz↓*	FP1↓*, FP2↓*, F3↓*, F4↓*, C4↓*, P3↓*, P4↓*, O2↓*, F7↓*, F8↓*, T6↓*, Cz↓*	F3↑*, F8↑*, T3↑*, T4↑*, T5↑*

Two-sided nonparametric Wilcoxon rank-sum tests were taken. MP and ACTH treat the brain network by reducing the overall transmission efficiency, local connectivity and defense ability of the fast waves.

* Indicates statistical significance at *P *< 0.05.

##### 3.3.2.3. During the waking ictal phase

**Figure 6:**
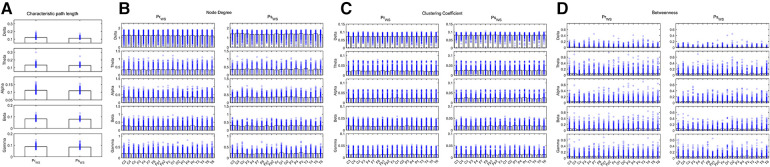
Network attributes in the IESS group during the waking ictal phase before and after hormonal therapy. (**A**): CPL. (**B**): ND (**C**): CC. (**D**): BC. PrWS: network attributes of IESS group during the waking ictal phase before hormonal therapy. PsWS: network attributes of the control group during the waking ictal phase after hormonal therapy.

During the waking ictal phase Post-hormonal treatment, the CPL of the Delta, Alpha, Beta, and Gamma bands decreased. The ND and CC increased in the Delta, Theta, Beta, and most leads of the Alpha, and the BC decreased in the Delta band comparing the IESS group with that before hormonal therapy(all *P* < 0.05) (as shown in Figure 6 and Table 5).

**Table 5:** Comparison of network attributes properties before and after hormonal therapy during the waking ictal phase.

Band	CPL	ND	CC	BC
Delta	↓*	Fp1↑*, Fp2↑*, F3↑*, F4↑*, C3↑*, C4↑*, P3↑*, P4↑*, O1↑*, O2↑*, F7↑*, F8↑*, T3↑*, T4↑*, T5↑*, T6↑*, Fz↑*, Cz↑*, Pz↑*	Fp1↑*, Fp2↑*, F3↑*, F4↑*, C3↑*, C4↑*, P3↑*, P4↑*, O1↑*, O2↑*, F7↑*, F8↑*, T3↑*, T4↑*, T5↑*, T6↑*, Fz↑*, Cz↑*, Pz↑*	Fp1↓*, Fp2↓*, F3↓*, F4↓*, C4↓*, P3↓*, O1↓*, O2↓*, F7↓*, T3↓*
Theta	–	F3↑*, F4↑*, C3↑*, P4↑*, O2↑*, F7↑*, F8↑*, T3↑*, T5↑*, T6↑*, Cz↑*	Fp2↑*, F3↑*, C3↑*, P4↑*, O1↑*, F7↑*, F8↑*, T4↑*, T5↑*, T6↑*, Cz↑*	Fp2↓*, F3↑*, F7↑*, F8↓*, T3↑*, T4↓*, Fz↓*, Cz↑*
Alpha	↓*	Fp2↑*, F3↑*, P4↑*, O1↑*, O2↑*, F7↑*, Fz↑*, Pz↓*	Fp2↑*, P3↑*, P4↑*, T4↓*, T5↓*, T6↑*, Pz↑*	Fp2↑*, C3↓*, O2↑*, F8↑*, Fz↑*, Pz↓*
Beta	↓*	Fp1↑, F3↑, C3↑, C4↑, P3↑, P4↑, O1↑, F7↑, T3↑, T4↑, T5↑, T6↑, Fz↑	Fp1↑*, P3↑*, P4↑*, F7↑*, T3↑*, T5↑*, T6↑*, Pz↑*	F3↑*, F4↓*, C3↑*, O1↑*, T4↑*, Fz↑*, Pz↓*
Gamma	↓*	Fp1↓*, Fp2↓*, F3↓*, F4↓*, O1↑*, O2↓*, T5↑*, T6↑*, Pz↑*	Fp1↓*, Fp2↓*, P4↓*, O1↓*, F7↑*, F8↑*	Fp1↓*, F3↓*, F4↓*, P3↓*, P4↑*, O2↓*, Pz↑*

Two-sided nonparametric Wilcoxon rank-sum tests were taken. MP and ACTH enhanced the fast waves’ overall transmission efficiency, local connectivity and defense capacity.

* Indicates statistical significance at *P *< 0.05.

##### 3.3.2.4. During the sleep ictal phase

**Figure 7:**
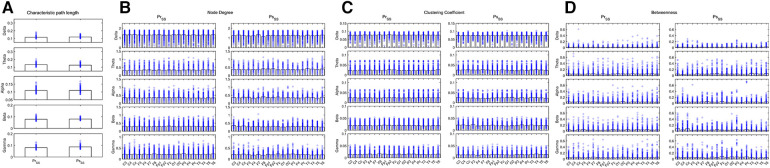
Network attributes in the IESS group during the sleep ictal phase before and after hormonal therapy. (**A**): CPL. (**B**): ND (**C**): CC. (**D**): BC. PrSS: network attributes of IESS group during the sleep ictal phase before hormonal therapy. PsSS: network attributes of the control group during the sleep ictal phase after hormonal therapy.

Comparing the IESS group before hormonal therapy during the sleep ictal phase, the CPL of the Delta band increased, but decreased for the Theta band after hormonal therapy. The ND for the Theta and Alpha bands increased, while it decreased for the Delta band after hormonal therapy. The CC decreased in the Delta band, and the BC of each frequency band primarily increased after hormonal therapy (all *P* < 0.05) (as shown in Figure 7 and Table 6).

**Table 6:** Comparison of network attributes before and after hormonal therapy during the sleep ictal phase.

Band	CPL	ND	CC	BC
Delta	↑*	Fp1↓*, F3↓*, F4↓*, C3↓*, P3↓*, O1↓*, F8↓*, T4↓*, T5↓*, Cz↓*, Pz↓*	Fp1↓*, Fp2↓*, F3↓*, F4↓*, C3↓*, C4↓*, P3↓*, P4↓*, O1↓*, O2↓*, F7↓*, F8↓*, T3↓*, T4↓*, T5↓*, T6↓*, Fz↓*, Pz↓*	Fp2↑*, F4↑*, C3↑*, C4↑*, P4↑*, O1↑*, O2↑*, F7↑*, T4↓*, T6↑*, Fz↑*
Theta	↓*	Fp1↑*, Fp2↑*, F3↑*, C3↑*, C4↑*, O2↑*, F7↑*, F8↑*, T3↑*, T4↑*, T6↑*, Cz↑*	Fp2↓*, C3↑*, C4↑*, P4↑*, F7↑*, T4↑*, T6↓*	Fp2↑*, F8↑*, T3↑*, T5↓*, T6↑*
Alpha	–	F3↑*, F8↑*, T3↑*, T4↑*, T6↑*, Cz↑*	F4↓*, P3↓*, P4↑*, O2↑*, T3↑*, T6↓*	P4↓*, O2↓*, F8↑*, T3↑*, T4↑*, T6↑*, Fz↑*, Cz↑*
Beta	–	C3↑*, O1↓*, T6↓*, Cz↑*	Fp2↓*, F3↑*, F4↓*, O1↓*, T3↑*, T4↑*	Fp1↑*, C3↑*, C4↑*, O1↓*, T4↓*, Cz↑*
Gamma	–	F4↓*, C3↓*, P3↓*, O1↓*, F8↓*, T4↓*, T5↓*, T6↓*, Fz↑*	Fp1↓*, F4↓*, C3↓*, O1↓*, O2↓*, F8↓*, T4↑*, Pz↓*	Fp1↑*, F4↑*, P4↑*, O2↑*, F8↓*, T4↓*, T6↓*, Fz↑*, Pz↑*

Two-sided nonparametric Wilcoxon rank-sum tests were taken. MP and ACTH played roles in reduced the overall transmission efficiency, local connectivity and defense capacity of the Delta band.

* Indicates statistical significance at *P *< 0.05.

## 4. Discussion

In our study, we primarily demonstrated that the CPL of the Delta band in children with IESS increased while ND and CC of the same band decreased during the waking period. However, these measures increased in the fast wave band. Conversely, during sleep, the CPL of the Delta band decreased, whereas the ND and CC of the Delta, Alpha, Beta, and Gamma bands increased. Identifying biomarkers from hypsarrhythmia on cranial EEG represents a development trend in the big data era.

Zhang et al. identified a significant difference in EEG complexity between patients with IESS and normal controls (14). Fast (40–150 Hz) oscillations in IESS have been associated with disorders involving structural brain pathology (15). Furthermore, Burroughs et al. found that neuronal networks are significantly altered in children with IESS, particularly during sleep (16). Our research indicated an enhancement in local connectivity and defense ability of fast waves in children with IESS during the wake-sleep period. We also discovered a decrease in overall transmission efficiency, local connectivity, and defense capacity of the Delta band during the waking period, contrasting with network attributes alternation during sleep, which could be explained by increased coherences due to cortico-subcortical circuitry synchronizing cortical activity (16). This enhanced brain network connectivity during sleep likely strengthens information transmission of network attributes with IESS, leading to hypsarrhythmia and seizure onset (12). These findings may manifest the network attributes characteristics of IESS.

IESS, updated in 2022 by The International League Against Epilepsy (ILAE) from IS, is a common intractable epilepsy disorder (1). The clinical manifestations of IESS were strings of or isolated spasms characterized by hypsarrhythmia on EEG. The BASED score, an interictal EEG grading scale revised in 2021, is an effective tool for identifying and diagnosing children with IESS (5, 17). According to our research, the BASED score of children with IESS was 4.83 ± 0.38 before treatment. Approximately 80% to 90% of children with IESS experience severe mental and motor delays or regression (18), consistent with our study (89.47%). This might be due to weakened connections between brain regions responsible for intelligence and cognition caused by recurrent seizures (19). The first-line treatment for IESS includes ACTH and vigabatrin (2, 20), and MP treatment for IESS proved to have the same effect as ACTH (21). In our study, the BASED score significantly decreased (3.44 ± 0.82 vs. 4.83 ± 0.38) after immediate hormone pulse therapy. A BASED-based scoring model constructed using convolutional neural networks achieved an impressive classification accuracy of 96% (17), but it failed to improve the inter-rater agreement of hypsarrhythmia (13). Considering the limited information from interictal EEG, there is still much to understand about network attributes for IESS. Additionally, it would be beneficial to identify alterations in hormone patterns to the brain networks.

Chu et al. found that total EEG complexity was associated with spasm freedom (6). In our study, we compared the brain network properties before and after hormonal therapy in the IESS group, finding that CPL of the fast waves increased and ND and CC decreased during the sleep-wake phase. Some studies have shown that occipital EEG complexities in the *γ* band optimize performance in identifying responses to treatment (14). Also, the connectivity of Theta over the whole brain in hypsarrhythmia might correlate with cortical epileptogenicity refractory to medical and surgical treatments (22). Douw et al. found that theta band synchronization likelihood was a significant predictor of epilepsy diagnosis (23). Partially in line with these findings, our results indicated a decrease in the ND and CC of the Theta band, implying that MP and ACTH could ameliorate brain network dysfunction in IESS children by reducing the overall transmission efficiency, local connectivity, and defense ability of the fast waves during interictal periods and the Theta band during the waking period. From our results, it can be inferred that the altered network attributes of the fast waves and Theta band can predict the effects of treatment early. These confirm the previous findings that the marked decrease in slope reflected a loss of synaptic connections due to the effect of glucocorticoids (24).

Lastly, we presented evidence that the local connectivity and defense capability of the slow waves were enhanced in patients with IESS during ictal onset (12). Notably, we observed that the CPL in the Delta band decreased, and ND and CC increased during the wake ictal phase, contrasting with the sleep ictal phase. This implies a therapeutic role for MP and ACTH in reducing the overall transmission efficiency, local connectivity, and defense capacity of the Delta band during the sleep ictal state. Our data uniquely demonstrate that hormones have different effects under different conditions.

Moreover, HFOs have been identified as potential triggers for spasms (25). Despite an in-depth understanding of the focal “leading” spike via intracranial electrocorticography and seizure onset zone localization by stereo-electroencephalography (SEEG) electrodes in IESS (26, 27), the thin skull bone of infants and the non-structural etiology associated with IESS pose relative contraindications for SEEG implantation (26).

Changes in *β* and *γ* frequency bands have been noted as indicators of seizure cycle changes in IESS (19). In addition, the analysis of average HFO energy could serve as a predictive measure for the effectiveness of epilepsy treatments (5). Our study found that following hormonal therapy, the CPL of the fast waves decreased while ND and CC increased during the wakeful ictal state. This led us to conclude that MP and ACTH could enhance the overall transmission efficiency, local connectivity, and defense capacity of the fast waves. From a network attributes perspective, it appears that enhanced information transmission capacity during the waking state might explain why IESS is not readily controlled over a short period. Hence, we propose a new explanation as to why seizure control has not been initially achieved.

Our research forms a new foundation for the development of diagnosis and treatment methods for IESS from a network attributes perspective. It offers resources to identify new candidates for a timely prognosis. However, this study is not without limitations. We sought to investigate and summarize the standard features of IESS, but overlooked the metabolic and infectious etiologies of IESS, which are infrequent in our practical setting. In future research, we aim to encompass and separately study patients with differing etiologies. Additionally, due to the sample size, the cases were not further subdivided according to treatment effect. Instead, we examined the global impact of hormone therapy on network attributes in children with IESS. By doing so, we aim to predict the effects of hormonal therapy on prognosis determination and timely adjustment of treatment regimens.

## 5. Conclusion

In conclusion, graph theory-based EEG analyses in IESS patients may serve as a promising avenue for biomarker identification. Our findings suggest that the fast waves and Delta band play a significant role in the brain network attributes of IESS. Concurrently, MP and ACTH can remediate the dysfunctions leading to the emergence of the fast waves and Theta band during the interictal period, and the Delta band during the ictal state. Collectively, these results offer a unique insight into the EEG biomarkers of IESS patients and elucidate the modes of action of hormones on network attributes.

## Data Availability

The original contributions presented in the study are included in the article/**Supplementary Material**, further inquiries can be directed to the corresponding author.

## References

[1] Zuberi SM Wirrell E Yozawitz E Wilmshurst JM Specchio N Riney K ILAE Classification and definition of epilepsy syndromes with onset in neonates and infants: position statement by the ILAE task force on nosology and definitions. Epilepsia. (2022) 63(6):1349–97. 10.1111/epi.17239 35503712

[2] Kelley SA Knupp KG. Infantile spasms-have we made progress? Curr Neurol Neurosci Rep. (2018) 18(5):27. 10.1007/s11910-018-0832-8 29671077

[3] Cohen AL Mulder BPF Prohl AK Soussand L Davis P Kroeck MR Tuber locations associated with infantile spasms map to a common brain network. Ann Neurol. (2021) 89(4):726–39. 10.1002/ana.26015 33410532 PMC7969435

[4] McCrimmon CM Riba A Garner C Maser AL Phillips DJ Steenari M Automated detection of ripple oscillations in long-term scalp EEG from patients with infantile spasms. J Neural Eng. (2021) 18(1):1–14. 10.1088/1741-2552/abcc7e 33217752

[5] Romero Milà B Remakanthakurup Sindhu K Mytinger JR Shrey DW Lopour BA. EEG biomarkers for the diagnosis and treatment of infantile spasms. Front Neurol. (2022) 13:960454. 10.3389/fneur.2022.960454 35968272 PMC9366674

[6] Chu Y-J Chang C-F Weng W-C Fan P-C Shieh J-S Lee W-T. Electroencephalography complexity in infantile spasms and its association with treatment response. Clin Neurophysiol. (2021) 132(2):480–6. 10.1016/j.clinph.2020.12.006 33450568

[7] Wang W Li H Yan J Zhang H Li X Zheng S Automatic detection of interictal ripples on scalp EEG to evaluate the effect and prognosis of ACTH therapy in patients with infantile spasms. Epilepsia. (2021) 62(9):2240–51. 10.1111/epi.17018 34309835

[8] Stacey W Kramer M Gunnarsdottir K Gonzalez-Martinez J Zaghloul K Inati S Emerging roles of network analysis for epilepsy. Epilepsy Res. (2020) 159:106255. 10.1016/j.eplepsyres.2019.106255 31855828 PMC6990460

[9] Soriano-Mas C. Functional brain imaging and OCD. Curr Top Behav Neurosci. (2021) 49:269–300. 10.1007/7854_2020_203 33604877

[10] Zhang Y Huang G Liu M Li M Wang Z Wang R Functional and structural connective disturbance of the primary and default network in patients with generalized tonic-clonic seizures. Epilepsy Res. (2021) 174:106595. 10.1016/j.eplepsyres.2021.106595 33993017

[11] Tsuchiya H Endoh F Akiyama T Matsuhashi M Kobayashi K. Longitudinal correspondence of epilepsy and scalp EEG fast (40–200 hz) oscillations in pediatric patients with tuberous sclerosis complex. Brain Dev. (2020) 42(9):663–74. 10.1016/j.braindev.2020.06.001 32631641

[12] Dong Y Xu R Zhang Y Shi Y Du K Jia T Different frequency bands in various regions of the brain play different roles in the onset and wake-sleep stages of infantile spasms. Front Pediatr. (2022) 10:878099. 10.3389/fped.2022.878099 35633963 PMC9135356

[13] Mytinger JR Vidaurre J Moore-Clingenpeel M Stanek JR Albert DVF. A reliable interictal EEG grading scale for children with infantile spasms—the 2021 BASED score. Epilepsy Res. (2021) 173:106631. 10.1016/j.eplepsyres.2021.106631 33839516

[14] Zhang C-T Sun Y-L Shi W-B Yang G Yeh C-H. Brain complexity predicts response to adrenocorticotropic hormone in infantile epileptic spasms syndrome: a retrospective study. Neurol Ther. (2023) 12(1):129–44. 10.1007/s40120-022-00412-1 36327095 PMC9837343

[15] Kobayashi K Endoh F Agari T Akiyama T Akiyama M Hayashi Y Complex observation of scalp fast (40–150 hz) oscillations in west syndrome and related disorders with structural brain pathology. Epilepsia Open. (2017) 2(2):260–6. 10.1002/epi4.12043 29588955 PMC5719855

[16] Burroughs SA Morse RP Mott SH Holmes GL. Brain connectivity in West syndrome. Seizure. (2014) 23(7):576–9. 10.1016/j.seizure.2014.03.016 24794162 PMC4361818

[17] Fan Y Chen D Wang H Pan Y Peng X Liu X Automatic BASED scoring on scalp EEG in children with infantile spasms using convolutional neural network. Front Mol Biosci. (2022) 9:931688. 10.3389/fmolb.2022.931688 36032671 PMC9399419

[18] Kvernadze A Tatishvili N Lomidze G Tarkhnishvili N Kipiani T Tatishvili S. Predictors of outcome among 31 children with infantile spasms syndrome. Epileptic Disord. (2022) 24(2):359–72. 10.1684/epd.2021.1397 34887239

[19] Zheng R Feng Y Wang T Cao J Wu D Jiang T Scalp EEG functional connection and brain network in infants with West syndrome. Neural Netw. (2022) 153:76–86. 10.1016/j.neunet.2022.05.029 35714423

[20] Ko A Youn SE Chung HJ Kim SH Lee JS Kim HD Vigabatrin and high-dose prednisolone therapy for patients with West syndrome. Epilepsy Res. (2018) 145:127–33. 10.1016/j.eplepsyres.2018.06.013 29966811

[21] Rajpurohit M Gupta A Madaan P Sahu JK Singhi P. Safety, feasibility and effectiveness of pulse methylprednisolone therapy in comparison with intramuscular adrenocorticotropic hormone in children with west syndrome. Indian J Pediatr. (2021) 88(7):663–7. 10.1007/s12098-020-03521-7 33103229

[22] Baba S Vakorin VA Doesburg SM Nagamori C Cortez MA Honda R EEG Before and after total corpus callosotomy for pharmacoresistant infantile spasms: fast oscillations and slow-wave connectivity in hypsarrhythmia. Epilepsia. (2019) 60(9):1849–60. 10.1111/epi.16295 31407333

[23] Douw L de Groot M van Dellen E Heimans JJ Ronner HE Stam CJ ‘Functional connectivity’ is a sensitive predictor of epilepsy diagnosis after the first seizure. PloS One. (2010) 5(5):e10839. 10.1371/journal.pone.0010839 20520774 PMC2877105

[24] Fattinger S Schmitt B Bölsterli Heinzle BK Critelli H Jenni OG Huber R. Impaired slow wave sleep downscaling in patients with infantile spasms. Eur J Paediatr Neurol. (2015) 19(2):134–42. 10.1016/j.ejpn.2014.11.002 25530030

[25] Yan L Li L Chen J Wang L Jiang L Hu Y. Application of high-frequency oscillations on scalp EEG in infant spasm: a prospective controlled study. Front Hum Neurosci. (2021) 15:682011. 10.3389/fnhum.2021.682011 34177501 PMC8223253

[26] Abel TJ Losito E Ibrahim GM Asano E Rutka JT. Multimodal localization and surgery for epileptic spasms of focal origin: a review. Neurosurg Focus. (2018) 45(3):E4. 10.3171/2018.6.FOCUS18217 30173609

[27] Zhang C Liu W Zhang J Zhang X Huang P Sun B Utility of magnetoencephalography combined with stereo-electroencephalography in resective epilepsy surgery: a 2-year follow-up. Seizure. (2022) 97:94–101. 10.1016/j.seizure.2022.03.013 35390641

