# Supplementary material for: Crucial involvement of fast waves and Delta band in the brain network attributes of infantile epileptic spasms syndrome
Source: Front Pediatr. 2023 Oct 20;11:1249789. doi: 10.3389/fped.2023.1249789 (PMC10623136; doi:10.3389/fped.2023.1249789)
Supplement: Supplementary file 1 [file Table1.pdf]

Supplementary-Table S1

Measure	denotation	Representation	Weighted and directed definitions
CPL	the average path length of all nodes in the network	information transmission of the whole-brain network	$L^W = \frac{1}{n}\sum_{i\in N}\frac{\sum_{j\in N, j\neq i} d_{ij}^w}{n-1}$
ND	the number of edges linked to a node	the local connectivity of the node in the local network	$k_i^w = \sum_{j\in N} wij$
CC	densely connected nodes in a network; the number of edges between its adjacent nodes divided by the theoretical maximum of edges these nodes could form	the local information transmission capacity of the network; defense capacity of the complex network	$C^w = \frac{1}{n}\sum_{i\in N}\frac{2t_i^w}{k_{i(k_i-1)}}$
BC	a nodes centrality in a network equal to the number of shortest paths between all pairs of nodes that pass through that node	node centrality from the perspective of information flow; the importance of the location of the nodes.	$C_b(i) = \sum_{j\neq i\neq k\in G}\frac{g_{jk}(i)}{g_{jk}}$

Supplementary-Table S2 (containing 12 tables)

All EEG data and statistical analyses were performed using MATLAB software (the MathWorks Inc., Natick, MA, USA). Brain functional network attributes were compared with two-sided non-parametric Wilcoxon rank-sum tests.

Table 1 The comparisons of CPL between the IESS and control group during the waking phase

Frequency	*Z*	*P*
Delta	-2.645	0.008
Theta	-1.549	0.121
Alpha	-0.155	0.877
Beta	-9.236	<0.001
Gamma	-3.282	0.001

CPL: Characteristic path length

Table 2 The comparisons of ND between the IESS and control group during the waking phase

Node	delta		theta		alpha		beta		gamma	
	Z	*P*	*Z*	*P*	*Z*	*P*	*Z*	*P*	*Z*	*P*
Fp1	-1.879	0.06	-0.013	0.99	-0.343	0.731	-8.209	<0.001	-1.769	0.077
Fp2	-2.187	0.029	-1.166	0.243	-0.233	0.816	-8.945	<0.001	-0.611	0.541
F3	-2.115	0.034	-1.004	0.315	-0.759	0.448	-7.431	<0.001	-1.089	0.276
F4	-3.553	<0.001	-1.836	0.066	-1.244	0.213	-8.883	<0.001	-0.709	0.478
C3	-3.373	0.001	-2.225	0.026	-1.929	0.054	-9.518	<0.001	-0.939	0.348
C4	-1.432	0.152	-2.59	0.01	-0.888	0.375	-8.531	<0.001	-1.188	0.235
P3	-2.486	0.013	-2.214	0.027	-0.911	0.362	-8.514	<0.001	-1.676	0.094
P4	-1.64	0.101	-1.781	0.075	-0.644	0.519	-8.783	<0.001	-0.457	0.648
O1	-2.908	0.004	-0.994	0.32	-3.199	0.001	-9.07	<0.001	-0.611	0.541
O2	-3.023	0.003	-0.064	0.949	-0.495	0.621	-7.467	<0.001	-2.192	0.028
F7	-0.156	0.876	-1.083	0.279	-0.78	0.435	-7.522	<0.001	-1.026	0.305
F8	-1.918	0.055	-0.564	0.573	-0.215	0.829	-8.391	<0.001	-0.071	0.944
T3	-0.496	0.62	-1.059	0.29	-1.787	0.074	-8.349	<0.001	-1.219	0.223
T4	-0.918	0.359	-0.184	0.854	-0.364	0.716	-8.415	<0.001	-0.405	0.685
T5	-2.577	0.01	-1.639	0.101	-2.895	0.004	-7.815	<0.001	-0.354	0.723
T6	-4.612	<0.001	-1.009	0.313	-0.456	0.649	-7.447	<0.001	-1.183	0.237
Fz	-1.409	0.159	-1.098	0.272	-1.036	0.3	-7.907	<0.001	-0.213	0.831
Cz	-1.153	0.249	-0.129	0.897	-1.575	0.115	-6.112	<0.001	-0.093	0.926

Pz	-2.066	0.039	-0.1	0.92	-0.076	0.94	-8.905	<0.001	-0.815	0.415

ND: Node degree

Table 3 The comparisons of CC between the IESS and control group during the waking phase

Node	delta		theta		alpha		beta		gamma	
	Z	*P*	*Z*	*P*	*Z*	*P*	*Z*	*P*	*Z*	*P*
Fp1	-1.843	0.065	-0.815	0.415	-1.364	0.173	-7.048	<0.001	-3.34	0.001
Fp2	-2.866	0.004	-0.449	0.653	-0.371	0.711	-9.222	<0.001	-0.016	0.987
F3	-2.228	0.026	-1.296	0.195	-0.61	0.542	-6.778	<0.001	-3.009	0.003
F4	-1.813	0.07	-1.244	0.214	-0.112	0.911	-7.302	<0.001	-1.199	0.231
C3	-3.004	0.003	-1.764	0.078	-0.162	0.871	-7.017	<0.001	-0.635	0.526
C4	-1.66	0.097	-0.404	0.686	-1.791	0.073	-8.154	<0.001	-1.625	0.104
P3	-2.402	0.016	-1.423	0.155	-0.782	0.434	-8.66	<0.001	-1.985	0.047
P4	-1.837	0.066	-1.456	0.145	-0.94	0.347	-6.325	<0.001	-0.346	0.729
O1	-2.496	0.013	-0.565	0.572	-0.676	0.499	-8.929	<0.001	-1.244	0.213
O2	-1.648	0.099	-0.866	0.387	-0.114	0.909	-6.785	<0.001	-1.769	0.077
F7	-1.594	0.111	-0.393	0.694	-1.388	0.165	-8.945	<0.001	-0.195	0.846
F8	-1.25	0.211	-0.738	0.46	-0.769	0.442	-9.106	<0.001	-2.036	0.042
T3	-3.746	<0.001	-0.373	0.709	-0.711	0.477	-6.12	<0.001	-0.009	0.993
T4	-1.678	0.093	-0.704	0.481	-1.588	0.112	-7.09	<0.001	-0.738	0.46
T5	-2.988	0.003	-2.034	0.042	-0.568	0.57	-8.347	<0.001	-0.221	0.825
T6	-0.211	0.833	-0.805	0.421	-0.288	0.773	-7.392	<0.001	-1.413	0.158
Fz	-3.038	0.002	-0.065	0.948	-0.951	0.342	-6.421	<0.001	-2.257	0.024
Cz	-1.561	0.119	-0.113	0.91	-0.323	0.747	-6.824	<0.001	-0.828	0.408
Pz	-3.244	0.001	-2.337	0.019	-0.453	0.651	-5.781	<0.001	-0.372	0.71

CC: Clustering coefficient

Table 4 The comparisons of BC between the IESS and control group during the waking phase

Node	delta		theta		alpha		beta		gamma	
	Z	*P*	*Z*	*P*	*Z*	*P*	*Z*	*P*	*Z*	*P*
Fp1	-0.539	0.590	-0.419	0.675	-1.244	0.214	-1.989	0.047	-0.287	0.774
Fp2	-1.194	0.232	-0.98	0.327	-0.601	0.548	-3.074	0.002	-0.939	0.348
F3	-0.264	0.792	-1.900	0.057	-1.042	0.297	-3.431	0.001	-2.429	0.015

F4	-0.887	0.375	-1.821	0.069	-2.679	0.007	-2.707	0.007	-0.002	0.999
C3	-0.082	0.935	-0.404	0.686	-0.136	0.892	-0.464	0.643	-1.234	0.217
C4	-0.987	0.324	-2.727	0.006	-1.145	0.252	-1.100	0.271	-0.318	0.751
P3	-0.938	0.348	-0.127	0.899	-0.892	0.372	-2.002	0.045	-0.344	0.731
P4	-2.132	0.033	-1.066	0.286	-0.159	0.874	-1.193	0.233	-1.926	0.054
O1	-1.342	0.180	-2.068	0.039	-2.019	0.043	-1.809	0.070	-0.098	0.922
O2	-0.171	0.864	-0.660	0.509	-0.810	0.418	-2.687	0.007	-0.306	0.760
F7	-0.602	0.547	-2.006	0.045	-0.266	0.791	-3.698	<0.001	-0.388	0.698
F8	-1.002	0.317	-0.376	0.707	-1.167	0.243	-1.955	0.051	-1.536	0.124
T3	-2.376	0.017	-0.568	0.570	-1.961	0.050	-0.132	0.895	-1.497	0.134
T4	-2.060	0.039	-0.519	0.604	-0.511	0.609	-0.587	0.557	-0.155	0.877
T5	-1.567	0.117	-0.212	0.832	-2.111	0.035	-1.786	0.074	-0.045	0.964
T6	-2.733	0.006	-0.948	0.343	-0.361	0.718	-2.291	0.022	-0.705	0.481
Fz	-1.727	0.084	-1.553	0.120	-1.519	0.129	-1.950	0.051	-1.465	0.143
Cz	-0.011	0.991	-0.175	0.861	-1.240	0.215	-3.153	0.002	-0.682	0.495
Pz	-1.499	0.134	-1.471	0.141	-0.342	0.732	-1.549	0.121	-0.267	0.790

BC：betweenness centrality

Table 5 The comparisons of CPL between the IESS and control group during the sleep phase

Frequency	*Z*	*P*
Delta	-2.688	0.007
Theta	-1.276	0.202
Alpha	-0.781	0.435

Beta	-5.079	<0.001
Gamma	-1.94	0.052

Table 6 The comparisons of ND between the IESS and control group during the sleep phase

Node	delta		theta		alpha		beta		gamma	
	Z	P	Z	P	Z	P	Z	P	Z	P
Fp1	-1.656	0.098	-1.712	0.087	-0.329	0.742	-4.374	<0.001	-2.513	0.012
Fp2	-2.777	0.005	-0.005	0.996	-0.142	0.887	-3.708	<0.001	-3.366	0.001
F3	-4.131	<0.001	-1.231	0.218	-2.419	0.016	-4.658	<0.001	-3.067	0.002
F4	-2.134	0.033	-2.04	0.041	-0.698	0.485	-2.416	0.016	-1.178	0.239
C3	-2.002	0.045	-3.103	0.002	-0.868	0.386	-3.641	<0.001	-2.29	0.022
C4	-0.785	0.433	-2.438	0.015	-1.62	0.105	-1.605	0.109	-2.777	0.005
P3	-0.688	0.492	-0.196	0.845	-1.054	0.292	-4.756	<0.001	-2.021	0.043
P4	-1.979	0.048	-1.68	0.093	-1.482	0.138	-4.064	<0.001	-2.823	0.005
O1	-0.475	0.635	-1.531	0.126	-1.36	0.174	-3.671	<0.001	-0.647	0.518
O2	-0.311	0.756	-2.398	0.016	-0.061	0.952	-5.256	<0.001	-1.034	0.301
F7	-2.019	0.044	-2.217	0.027	-1.807	0.071	-2.863	0.004	-1.817	0.069
F8	-3.452	0.001	-1.266	0.205	-1.152	0.249	-4.037	<0.001	-0.121	0.903
T3	-2.272	0.023	-0.225	0.822	-2.052	0.04	-4.924	<0.001	-0.964	0.335
T4	-2.98	0.003	-2.102	0.036	-0.985	0.325	-4.615	<0.001	-1.443	0.149
T5	-0.718	0.473	-1.515	0.13	-0.913	0.361	-6.443	<0.001	-1.086	0.277
T6	-1.526	0.127	-0.254	0.8	-0.849	0.396	-7.151	<0.001	-0.597	0.55
Fz	-2.813	0.005	-0.639	0.523	-0.051	0.96	-1.277	0.202	-1.445	0.149
Cz	-2.255	0.024	-1.669	0.095	-0.425	0.67	-0.888	0.375	-1.726	0.084
Pz	-0.553	0.58	-1.634	0.102	-0.464	0.643	-3.285	0.001	-2.518	0.012

Table 7 The comparisons of CC between the IESS and control group during the sleep phase

Node	delta		theta		alpha		beta		gamma	
	Z	P	Z	P	Z	P	Z	P	Z	P
Fp1	-3.676	<0.001	-1.388	0.165	-1.286	0.198	-4.667	<0.001	-4.588	<0.001
Fp2	-2.327	0.02	-0.531	0.595	-1.05	0.294	-3.475	0.001	-3.328	0.001
F3	-1.99	0.047	-0.964	0.335	-1.969	0.049	-3.491	<0.001	-5.163	<0.001
F4	-2.732	0.006	-1.243	0.214	-1.338	0.181	-2.808	0.005	-3.513	<0.001
C3	-1.993	0.046	-1.346	0.178	-0.876	0.381	-4.55	<0.001	-1.639	0.101
C4	-2.436	0.015	-1.041	0.298	-1.754	0.079	-3.168	0.002	-2.861	0.004

P3	-3.872	<0.001	-0.303	0.762	-0.823	0.41	-4.467	<0.001	-2.749	0.006
P4	-1.819	0.069	-0.789	0.43	-0.631	0.528	-3.906	<0.001	-3.97	<0.001
O1	-2.884	0.004	-2.536	0.011	-0.841	0.4	-4.079	<0.001	-3.001	0.003
O2	-2.265	0.024	-0.884	0.377	-1.64	0.101	-4.294	<0.001	-1.827	0.068
F7	-2.411	0.016	-0.721	0.471	-0.14	0.889	-3.92	<0.001	-2.014	0.044
F8	-1.464	0.143	-1.082	0.279	-0.323	0.747	-2.639	0.008	-2.065	0.039
T3	-2.355	0.019	-0.41	0.682	-0.525	0.599	-3.938	<0.001	-1.765	0.078
T4	-3.438	0.001	-1.009	0.313	-1.439	0.15	-4.932	<0.001	-1.867	0.062
T5	-2.948	0.003	-0.609	0.542	-0.234	0.815	-4.12	<0.001	-1.944	0.052
T6	-2.779	0.005	-1.586	0.113	-0.681	0.496	-4.233	<0.001	-3.199	0.001
Fz	-1.676	0.094	-0.45	0.653	-0.818	0.413	-1.494	0.135	-2.744	0.006
Cz	-2.183	0.029	-1.264	0.206	-0.148	0.882	-3.292	0.001	-2.596	0.009
Pz	-3.6	<0.001	-0.365	0.715	-0.706	0.48	-2.979	0.003	-2.248	0.025

Table 8 The comparisons of BC between the IESS and control group during the sleep phase

Node	delta		theta		alpha		beta		gamma	
	Z	*P*	*Z*	*P*	*Z*	*P*	*Z*	*P*	*Z*	*P*
Fp1	-2.906	0.004	-2.317	0.02	-2.989	0.003	-0.593	0.553	-1.114	0.265
Fp2	-0.273	0.785	-0.234	0.815	-0.054	0.957	-1.879	0.06	-0.576	0.565
F3	-0.848	0.397	-0.789	0.43	-0.967	0.334	-0.34	0.734	-0.802	0.422
F4	-0.13	0.897	-0.831	0.406	-0.026	0.979	-2.927	0.003	-2.133	0.033
C3	-0.192	0.848	-2.347	0.019	-0.528	0.597	-1.47	0.141	-0.215	0.829
C4	-1.8	0.072	-1.847	0.065	-1.124	0.261	-2.082	0.037	-0.376	0.707
P3	-2.467	0.014	-0.012	0.991	-2.008	0.045	-1.067	0.286	-1.164	0.244
P4	-2.605	0.009	-0.582	0.56	-1.429	0.153	-0.309	0.757	-0.083	0.934
O1	-1.251	0.211	-0.337	0.736	-3.078	0.002	-2.892	0.004	-0.975	0.33
O2	-1.388	0.165	-0.76	0.447	-1.512	0.131	-0.603	0.547	-1.496	0.135
F7	-0.567	0.57	-0.247	0.805	-0.745	0.456	-1.292	0.196	-0.564	0.573
F8	-1.552	0.121	-0.938	0.348	-0.913	0.361	-0.056	0.956	-1.858	0.063
T3	-0.95	0.342	-0.939	0.348	-0.244	0.807	-0.404	0.686	-1.507	0.132
T4	-0.25	0.802	-1.67	0.095	-1.775	0.076	-0.52	0.603	-4.285	<0.001
T5	-0.596	0.551	-1.274	0.203	-0.746	0.456	-1.764	0.078	-1.146	0.252
T6	-0.92	0.357	-0.294	0.769	-0.763	0.445	-2.57	0.01	-0.812	0.417
Fz	-0.272	0.786	-0.497	0.619	-1.378	0.168	-2.584	0.01	-0.969	0.332
Cz	-0.915	0.36	-0.929	0.353	-1.087	0.277	-3.874	<0.001	-0.705	0.481
Pz	-3.171	0.002	-0.886	0.376	-0.367	0.713	-1.208	0.227	-0.56	0.575

Table 9 The comparisons of CPL between before and after hormonal therapy in the IESS group during the waking phase

Frequency	Z	P
Delta	-0.353	0.724
Theta	-0.109	0.913
Alpha	-0.271	0.787
Beta	-4.503	<0.001
Gamma	-3.611	<0.001

Table 10 The comparisons of ND between before and after hormonal therapy in the IESS group during the waking phase

Node	delta		theta		alpha		beta		gamma	
	Z	*P*	*Z*	*P*	*Z*	*P*	*Z*	*P*	*Z*	*P*
Fp1	-2.451	0.014	-0.882	0.378	-0.896	0.37	-3.843	<0.001	-2.327	0.02
Fp2	-0.866	0.387	-0.264	0.792	-2.281	0.023	-3.504	<0.001	-0.721	0.471
F3	-1.463	0.143	-0.088	0.93	-1.797	0.072	-1.343	0.179	-2.629	0.009
F4	-0.735	0.462	-0.477	0.633	-0.408	0.684	-1.624	0.104	-0.957	0.338
C3	-0.841	0.4	-0.344	0.731	-0.361	0.718	-2.286	0.022	-1.726	0.084
C4	-0.079	0.937	-0.276	0.783	-0.335	0.738	-2.123	0.034	-1.632	0.103
P3	-0.331	0.741	-1.816	0.069	-0.829	0.407	-2.536	0.011	-2.121	0.034
P4	-0.567	0.571	-0.324	0.746	-2.186	0.029	-2.116	0.034	-1.647	0.099
O1	-0.182	0.855	-0.843	0.399	-2.439	0.015	-2.59	0.01	-1.93	0.054
O2	-0.291	0.771	-1.313	0.189	-0.757	0.449	-2.193	0.028	-1.707	0.088
F7	-0.562	0.574	-0.211	0.833	-0.045	0.964	-3.755	<0.001	-2.792	0.005
F8	-1.87	0.062	-0.578	0.563	-0.059	0.953	-2.157	0.031	-0.504	0.615
T3	-0.131	0.896	-0.619	0.536	-3.597	<0.001	-3.422	0.001	-2.305	0.021
T4	-0.166	0.868	-0.468	0.64	-0.423	0.672	-2.582	0.01	-0.031	0.975

T5	-1.089	0.276	-0.514	0.607	-2.086	0.037	-2.083	0.037	-2.55	0.011
T6	-0.706	0.48	-1.246	0.213	-0.323	0.747	-1.683	0.092	-3.381	0.001
Fz	-0.119	0.905	-2.252	0.024	-0.792	0.428	-3.778	<0.001	-1.423	0.155
Cz	-0.121	0.904	-1.164	0.244	-0.585	0.559	-2.501	0.012	-2.614	0.009
Pz	-1.284	0.199	-1.218	0.223	-0.346	0.729	-3.282	0.001	-1.627	0.104

Table 11 The comparisons of CC between before and after hormonal therapy in the IESS group during the waking phase

Node	delta		theta		alpha		beta		gamma	
	Z	*P*	*Z*	*P*	*Z*	*P*	*Z*	*P*	*Z*	*P*
Fp1	-1.897	0.058	-1.682	0.093	-3.057	0.002	-3.979	<0.001	-3.222	0.001
Fp2	-0.746	0.456	-0.461	0.645	-2.342	0.019	-5.086	<0.001	-3.14	0.002
F3	-0.581	0.561	-2.513	0.012	-0.81	0.418	-3.559	<0.001	-3.385	0.001
F4	-0.166	0.868	-2.063	0.039	-3.426	0.001	-3.883	<0.001	-4.256	<0.001
C3	-1.826	0.068	-0.757	0.449	-0.44	0.66	-2.204	0.028	-1.878	0.06
C4	-0.318	0.751	-1.152	0.249	-0.288	0.773	-3.177	0.001	-2.092	0.036
P3	-0.044	0.965	-0.738	0.46	-0.188	0.851	-3.428	0.001	-4.899	<0.001
P4	-1.027	0.304	-0.178	0.859	-0.541	0.588	-2.858	0.004	-3.177	0.001
O1	-0.131	0.896	-0.978	0.328	-2.431	0.015	-4.495	<0.001	-2.439	0.015
O2	-0.066	0.947	-1.97	0.049	-1.751	0.08	-2.804	0.005	-3.61	<0.001
F7	-0.395	0.693	-0.077	0.939	-1.089	0.276	-4.019	<0.001	-3.599	<0.001
F8	-0.627	0.53	-0.987	0.324	-2.913	0.004	-3.434	0.001	-4.562	<0.001
T3	-0.227	0.82	-0.689	0.491	-1.112	0.266	-2.508	0.012	-2.044	0.041
T4	-0.389	0.697	-0.494	0.621	-0.264	0.792	-2.949	0.003	-2.105	0.035
T5	-0.929	0.353	-0.16	0.873	-2.417	0.016	-4.804	<0.001	-3.197	0.001
T6	-0.743	0.457	-0.282	0.778	-1.021	0.307	-3.725	<0.001	-2.186	0.029
Fz	-0.385	0.7	-0.735	0.462	-1.851	0.064	-3.709	<0.001	-3.797	<0.001
Cz	-0.332	0.74	-0.304	0.761	-0.281	0.779	-2.647	0.008	-2.79	0.005
Pz	-1.16	0.246	-1.268	0.205	-2.742	0.006	-2.66	0.008	-2.8	0.005

Table 12 The comparisons of BC between before and after hormonal therapy in the IESS group during the waking phase

Node	delta		theta		alpha		beta		gamma	
	Z	*P*	*Z*	*P*	*Z*	*P*	*Z*	*P*	*Z*	*P*
Fp1	-2.642	0.008	-0.813	0.416	-2.493	0.013	-0.04	0.968	-1.707	0.088
Fp2	-2.128	0.033	-0.14	0.889	-0.146	0.884	-2.715	0.007	-2.558	0.011
F3	-1.296	0.195	-0.512	0.608	-2.767	0.006	-2.882	0.004	-1.35	0.177
F4	-2.685	0.007	-2.334	0.02	-3.03	0.002	-5.111	<0.001	-3.513	<0.001
C3	-1.06	0.289	-0.969	0.332	-0.395	0.693	-1.139	0.255	-0.385	0.7
C4	-0.216	0.829	-1.978	0.048	-0.541	0.588	-3.325	0.001	-0.791	0.429
P3	-0.707	0.48	-2.858	0.004	-0.524	0.6	-1.904	0.057	-1.851	0.064
P4	-1.945	0.052	-1.92	0.055	-3.153	0.002	-2.118	0.034	-1.181	0.238

O1	-0.692	0.489	-0.468	0.639	-0.893	0.372	-1.966	0.049	-0.978	0.328
O2	-0.343	0.732	-0.547	0.585	-0.051	0.96	-1.86	0.063	-1.675	0.094
F7	-2.88	0.004	-1.379	0.168	-0.572	0.567	-1.219	0.223	-0.328	0.743
F8	-1.408	0.159	-1.419	0.156	-2.863	0.004	-2.809	0.005	-2.95	0.003
T3	-1.208	0.227	-0.812	0.417	-2.248	0.025	-0.416	0.677	-0.772	0.44
T4	-1.009	0.313	-0.653	0.514	-1.014	0.311	-0.834	0.404	-1.266	0.205
T5	-0.623	0.533	-2.015	0.044	-2.103	0.035	-1.938	0.053	-0.08	0.936
T6	-0.32	0.749	-0.705	0.481	-0.964	0.335	-2.497	0.013	-1.163	0.245
Fz	-0.737	0.461	-1.543	0.123	-3.09	0.002	-0.162	0.871	-2.373	0.018
Cz	-2.042	0.041	-0.927	0.354	-0.39	0.697	-0.758	0.449	-0.014	0.989
Pz	-1.387	0.165	-0.391	0.696	-2.023	0.043	-1.24	0.215	-0.565	0.572

Table 13 The comparisons of CPL between before and after hormonal therapy in the IESS group during the sleep phase

Frequency	*Z*	*P*
Delta	-0.378	0.705
Theta	-1.482	0.138
Alpha	-0.544	0.586
Beta	-7.311	<0.001
Gamma	-1.552	0.121

Table 14 The comparisons of ND between before and after hormonal therapy in the IESS group during the sleep phase

Node	delta		theta		alpha		beta		gamma	
	Z	*P*	*Z*	*P*	*Z*	*P*	*Z*	*P*	*Z*	*P*
Fp1	-0.836	0.403	-2.117	0.034	-1.036	0.3	-5.793	<0.001	-2.063	0.039
Fp2	-0.38	0.704	-0.08	0.936	-0.41	0.682	-5.886	<0.001	-1.925	0.054
F3	-0.998	0.318	-0.471	0.638	-1.172	0.241	-4.808	<0.001	-0.111	0.911
F4	-1.025	0.305	-0.216	0.829	-0.01	0.992	-4.588	<0.001	-1.152	0.249
C3	-0.529	0.597	-1.814	0.07	-0.309	0.757	-4.194	<0.001	-1.056	0.291
C4	-1.343	0.179	-1.372	0.17	-0.443	0.658	-3.866	<0.001	-2.852	0.004
P3	-0.654	0.513	-0.813	0.416	-0.818	0.414	-6.63	<0.001	-1.146	0.252
P4	-0.141	0.888	-2.388	0.017	-0.187	0.852	-5.16	<0.001	-0.953	0.341
O1	-0.578	0.563	-1.594	0.111	-1.141	0.254	-5.542	<0.001	-0.752	0.452
O2	-0.424	0.672	-1.659	0.097	-1.459	0.144	-5.109	<0.001	-1.134	0.257
F7	-1.069	0.285	-0.557	0.578	-0.98	0.327	-4.493	<0.001	-1.952	0.051
F8	-1.174	0.24	-0.612	0.541	-0.56	0.576	-5.756	<0.001	-0.049	0.961

T3	-2.433	0.015	-0.487	0.627	-1.24	0.215	-4.683	<0.001	-0.258	0.796
T4	-1.152	0.249	-1.618	0.106	-0.036	0.971	-6.33	<0.001	-0.129	0.898
T5	-0.251	0.802	-0.559	0.576	-0.997	0.319	-6.88	<0.001	-1.139	0.255
T6	-0.533	0.594	-2.344	0.019	-1.161	0.246	-5.611	<0.001	-2.35	0.019
Fz	-0.861	0.389	-0.424	0.671	-0.348	0.728	-6.593	<0.001	-0.883	0.377
Cz	-0.684	0.494	-1.985	0.047	-0.95	0.342	-4.824	<0.001	-2.389	0.017
Pz	-0.618	0.537	-1.946	0.052	-2.023	0.043	-5.13	<0.001	-1.694	0.09

Table 15 The comparisons of CC between before and after hormonal therapy in the IESS group during the sleep phase

Node	delta		theta		alpha		beta		gamma	
	Z	*P*	*Z*	*P*	*Z*	*P*	*Z*	*P*	*Z*	*P*
Fp1	-3.074	0.002	-1.45	0.147	-3.313	0.001	-8.1	<0.001	-3.871	<0.001
Fp2	-2.303	0.021	-0.116	0.907	-1.324	0.186	-6.176	<0.001	-2.31	0.021
F3	-1.447	0.148	-0.419	0.675	-0.304	0.761	-4.985	<0.001	-2.331	0.02
F4	-1.713	0.087	-0.315	0.753	-1.228	0.219	-7.313	<0.001	-3.14	0.002
C3	-1.735	0.083	-2.908	0.004	-0.86	0.39	-3.467	0.001	-1.396	0.163
C4	-0.333	0.739	-0.883	0.377	-0.311	0.756	-4.467	<0.001	-3.264	0.001
P3	-1.702	0.089	-0.352	0.725	-1.194	0.232	-5.879	<0.001	-3.294	0.001
P4	-2.303	0.021	-0.627	0.53	-0.961	0.336	-5.642	<0.001	-2.784	0.005
O1	-0.895	0.371	-1.63	0.103	-0.833	0.405	-4.615	<0.001	-1.501	0.133
O2	-1.241	0.215	-0.563	0.573	-0.356	0.722	-6.839	<0.001	-3.14	0.002
F7	-0.9	0.368	-0.539	0.59	-0.919	0.358	-4.723	<0.001	-3.638	<0.001
F8	-0.306	0.76	-0.438	0.662	-0.022	0.983	-6.613	<0.001	-3.096	0.002
T3	-0.527	0.598	-1.551	0.121	-0.604	0.546	-4.162	<0.001	-1.262	0.207
T4	-1.654	0.098	-1.507	0.132	-1.085	0.278	-5.084	<0.001	-1.094	0.274
T5	-1.774	0.076	-0.86	0.39	-0.769	0.442	-5.402	<0.001	-0.816	0.415
T6	-1.141	0.254	-2.896	0.004	-0.884	0.377	-5.187	<0.001	-2.616	0.009
Fz	-0.31	0.756	-0.573	0.567	-1.176	0.239	-2.957	0.003	-1.06	0.289
Cz	-1.413	0.158	-2.384	0.017	-1.43	0.153	-5.538	<0.001	-2.757	0.006
Pz	-2.342	0.019	-0.778	0.437	-0.37	0.711	-6.823	<0.001	-1.63	0.103

Table 16 The comparisons of BC between before and after hormonal therapy in the IESS group during the sleep phase

Node	delta		theta		alpha		beta		gamma	
	Z	P	Z	P	Z	P	Z	P	Z	P
Fp1	-3.133	0.002	-2.588	0.01	-1.91	0.056	-3.701	<0.001	-0.5	0.617
Fp2	-2.666	0.008	-0.442	0.659	-0.522	0.602	-3.602	<0.001	-1.771	0.077
F3	-0.135	0.893	-1.08	0.28	-0.947	0.344	-2.67	0.008	-2.431	0.015
F4	-0.672	0.502	-0.465	0.642	-0.071	0.943	-4.627	<0.001	-0.873	0.383
C3	-0.871	0.384	-0.227	0.82	-0.234	0.815	-1.901	0.057	-0.911	0.363
C4	-1.159	0.246	-1.556	0.12	-0.622	0.534	-3.363	0.001	-0.601	0.548

P3	-1.081	0.28	-1.088	0.277	-0.508	0.611	-1.23	0.219	-1.11	0.267
P4	-1.474	0.14	-2.559	0.01	-1.275	0.202	-1.851	0.064	-1.861	0.063
O1	-0.601	0.548	-0.402	0.688	-1.574	0.115	-1.852	0.064	-0.129	0.897
O2	-0.976	0.329	-0.672	0.501	-0.113	0.91	-1.982	0.047	-1.089	0.276
F7	-0.515	0.607	-0.969	0.332	-0.474	0.636	-1.072	0.284	-0.796	0.426
F8	-1.796	0.073	-0.053	0.958	-0.723	0.47	-1.751	0.08	-2.798	0.005
T3	-1.376	0.169	-0.37	0.712	-1.327	0.185	-3.115	0.002	-1.959	0.05
T4	-0.585	0.559	-0.096	0.924	-0.655	0.513	-0.664	0.507	-2.322	0.02
T5	-0.276	0.782	-0.561	0.575	-0.442	0.659	-1.134	0.257	-3.342	0.001
T6	-1.593	0.111	-1.197	0.231	-1.481	0.139	-0.847	0.397	-0.197	0.844
Fz	-2.13	0.033	-2.153	0.031	-1.045	0.296	-1.636	0.102	-0.922	0.357
Cz	-0.814	0.415	-0.067	0.946	-0.941	0.347	-1.953	0.051	-0.648	0.517
Pz	-2.319	0.02	-1.44	0.15	-1.558	0.119	-2.473	0.013	-0.636	0.525

Table 17 The comparisons of CPL between before and after hormonal therapy in the IESS group during the waking ictal phase

Frequency	*Z*	*P*
Delta	-8.934	<0.001
Theta	-1.315	0.188
Alpha	-2.237	0.025
Beta	-4.804	<0.001
Gamma	-1.97	0.049

Table 18 The comparisons of ND between before and after hormonal therapy in the IESS group during the waking ictal phase

Node	delta		theta		alpha		beta		gamma	
	Z	*P*	*Z*	*P*	*Z*	*P*	*Z*	*P*	*Z*	*P*
Fp1	-5.231	<0.001	-1.351	0.177	-1.181	0.238	-3.841	<0.001	-2.498	0.013
Fp2	-5.166	<0.001	-0.619	0.536	-2.271	0.023	-0.582	0.561	-3.098	0.002
F3	-5.667	<0.001	-4.545	<0.001	-2.442	0.015	-4.454	<0.001	-2.291	0.022
F4	-5.268	<0.001	-2.232	0.026	-1.359	0.174	-1.484	0.138	-3.544	<0.001
C3	-6.392	<0.001	-2.494	0.013	-0.625	0.532	-3.962	<0.001	-0.48	0.631
C4	-10.321	<0.001	-1.925	0.054	-1.9	0.057	-2.844	0.004	-0.976	0.329
P3	-8.963	<0.001	-1.289	0.198	-1.558	0.119	-4.298	<0.001	-1.771	0.077
P4	-6.32	<0.001	-6.212	<0.001	-2.559	0.01	-2.497	0.013	-0.014	0.988
O1	-7.258	<0.001	-0.463	0.643	-2.076	0.038	-4.084	<0.001	-2.909	0.004

O2	-6.326	<0.001	-3.601	<0.001	-2.429	0.015	-1.478	0.139	-2.264	0.024
F7	-5.81	<0.001	-5.386	<0.001	-2.443	0.015	-2.012	0.044	-0.998	0.318
F8	-4.859	<0.001	-2.291	0.022	-1.69	0.091	-0.497	0.619	-0.402	0.688
T3	-7.332	<0.001	-4.105	<0.001	-0.476	0.634	-3.387	0.001	-0.09	0.928
T4	-4.003	<0.001	-1.7	0.089	-0.273	0.785	-4.149	<0.001	-0.868	0.385
T5	-9.458	<0.001	-4.3	<0.001	-1.292	0.196	-3.28	0.001	-2.483	0.013
T6	-4.911	<0.001	-4.043	<0.001	-1.459	0.145	-4.357	<0.001	-1.979	0.048
Fz	-7.969	<0.001	-1.433	0.152	-2.394	0.017	-3.293	0.001	-0.192	0.848
Cz	-8.558	<0.001	-3.561	<0.001	-0.323	0.747	-0.836	0.403	-0.878	0.38
Pz	-7.492	<0.001	-1.899	0.058	-2.54	0.011	-1.295	0.195	-4.465	<0.001

Table 19 The comparisons of CC between before and after hormonal therapy in the IESS group during the waking ictal phase

Node	delta		theta		alpha		beta		gamma	
	Z	*P*	*Z*	*P*	*Z*	*P*	*Z*	*P*	*Z*	*P*
Fp1	-9.125	<0.001	-0.446	0.655	-1.459	0.145	-2.66	0.008	-2	0.046
Fp2	-9.626	<0.001	-2.468	0.014	-2.465	0.014	-0.442	0.659	-2.279	0.023
F3	-8.3	<0.001	-3.504	<0.001	-1.294	0.196	-0.058	0.953	-0.278	0.781
F4	-6.872	<0.001	-1.441	0.15	-1.44	0.15	-0.912	0.362	-0.736	0.462
C3	-8.759	<0.001	-3.001	0.003	-0.007	0.994	-0.743	0.457	-0.713	0.476
C4	-6.972	<0.001	-1.137	0.255	-1.015	0.31	-1.248	0.212	-0.407	0.684
P3	-5.625	<0.001	-1.04	0.298	-2.161	0.031	-2.651	0.008	-0.924	0.355
P4	-8.874	<0.001	-4.978	<0.001	-1.988	0.047	-2.269	0.023	-3.053	0.002
O1	-6.908	<0.001	-2.307	0.021	-0.236	0.814	-1.681	0.093	-3.251	0.001
O2	-8.066	<0.001	-1.76	0.078	-1.519	0.129	-0.039	0.969	-1.186	0.236
F7	-8.352	<0.001	-3.266	0.001	-1.303	0.193	-2.862	0.004	-2.078	0.038
F8	-8.564	<0.001	-3.549	<0.001	-0.102	0.919	-0.111	0.912	-3.008	0.003
T3	-6.688	<0.001	-1.636	0.102	-1.368	0.171	-3.843	<0.001	-1.067	0.286
T4	-7.375	<0.001	-2.258	0.024	-2.701	0.007	-1.538	0.124	-0.286	0.775
T5	-6.299	<0.001	-6.077	<0.001	-2.046	0.041	-3.673	<0.001	-0.185	0.853
T6	-7.752	<0.001	-2.036	0.042	-4.035	<0.001	-3.045	0.002	-1.284	0.199
Fz	-7.468	<0.001	-1.92	0.055	-1.776	0.076	-0.966	0.334	-0.024	0.981
Cz	-4.744	<0.001	-2.246	0.025	-1.706	0.088	-0.59	0.555	-0.973	0.33
Pz	-6.705	<0.001	-1.922	0.055	-4.43	<0.001	-4.112	<0.001	-1.259	0.208

Table 20 The comparisons of BC between before and after hormonal therapy in the IESS group during the waking ictal phase

Node	delta		theta		alpha		beta		gamma	
	Z	*P*	*Z*	*P*	*Z*	*P*	*Z*	*P*	*Z*	*P*
Fp1	-5.664	<0.001	-1.359	0.174	-1.186	0.236	-0.677	0.498	-2.443	0.015
Fp2	-3.052	0.002	-3.278	0.001	-2.889	0.004	-1.859	0.063	-0.703	0.482
F3	-2.366	0.018	-2.008	0.045	-0.126	0.899	-2.706	0.007	-3.226	0.001

F4	-2.253	0.024	-1.85	0.064	-1.841	0.066	-2.454	0.014	-3.263	0.001
C3	-0.392	0.695	-0.168	0.867	-2.718	0.007	-3.24	0.001	-0.097	0.923
C4	-2.248	0.025	-0.123	0.902	-0.181	0.857	-0.715	0.475	-1.67	0.095
P3	-3.113	0.002	-1.828	0.068	-0.607	0.544	-1.222	0.222	-4.043	<0.001
P4	-1.581	0.114	-1.021	0.307	-1.117	0.264	-1.391	0.164	-2.636	0.008
O1	-6.264	<0.001	-1.117	0.264	-1.929	0.054	-2.043	0.041	-0.133	0.894
O2	-2.845	0.004	-0.657	0.511	-2.688	0.007	-0.997	0.319	-3.393	0.001
F7	-3.285	0.001	-2.353	0.019	-0.677	0.498	-1.303	0.193	-1.104	0.27
F8	-1.867	0.062	-1.98	0.048	-2.438	0.015	-1.518	0.129	-1.925	0.054
T3	-4.568	<0.001	-3.094	0.002	-0.162	0.872	-1.504	0.133	-0.003	0.997
T4	-1.565	0.118	-4.485	<0.001	-1.704	0.088	-1.967	0.049	-0.297	0.766
T5	-1.51	0.131	-0.145	0.885	-0.266	0.79	-0.238	0.812	-1.017	0.309
T6	-0.205	0.838	-0.236	0.813	-1.068	0.285	-0.775	0.438	-0.088	0.93
Fz	-0.04	0.968	-2.354	0.019	-2.398	0.016	-4.171	<0.001	-1.433	0.152
Cz	-0.892	0.372	-2.345	0.019	-0.404	0.686	-0.732	0.464	-1.641	0.101
Pz	-1.227	0.22	-1.506	0.132	-3.872	<0.001	-4.149	<0.001	-3.372	0.001

Table 21 The comparisons of CPL between before and after hormonal therapy in the IESS group during the sleep ictal phase

Frequency	*Z*	*P*
Delta	-4.412	<0.001
Theta	-6.109	<0.001
Alpha	-0.74	0.459
Beta	-0.826	0.409
Gamma	-1.506	0.132

Table 22 The comparisons of ND between before and after hormonal therapy in the IESS group during the sleep ictal phase

Node	delta		theta		alpha		beta		gamma	
	Z	*P*	*Z*	*P*	*Z*	*P*	*Z*	*P*	*Z*	*P*
Fp1	-4.068	<0.001	-3.786	<0.001	-0.148	0.883	-0.726	0.468	-0.145	0.885
Fp2	-1.505	0.132	-5.102	<0.001	-0.504	0.614	-1.749	0.08	-1.241	0.215
F3	-4.387	<0.001	-3.106	0.002	-3.014	0.003	-1.579	0.114	-1.439	0.15
F4	-3.245	0.001	-1.593	0.111	-1.281	0.2	-0.904	0.366	-5.068	<0.001
C3	-3.062	0.002	-3.974	<0.001	-0.962	0.336	-3.548	<0.001	-1.962	0.05
C4	-0.685	0.493	-3.988	<0.001	-0.187	0.852	-1.918	0.055	-0.515	0.607
P3	-2.076	0.038	-1.854	0.064	-1.039	0.299	-1.062	0.288	-2.209	0.027
P4	-1.384	0.166	-1.655	0.098	-0.077	0.938	-0.036	0.971	-0.249	0.803

O1	-2.698	0.007	-1.046	0.295	-0.504	0.614	-3.001	0.003	-1.958	0.05
O2	-1.881	0.06	-2.649	0.008	-1.362	0.173	-0.774	0.439	-0.161	0.872
F7	-0.854	0.393	-2.805	0.005	-0.448	0.654	-1.306	0.191	-0.098	0.922
F8	-7.169	<0.001	-6.259	<0.001	-3.293	0.001	-1.686	0.092	-6.351	<0.001
T3	-0.602	0.547	-6.927	<0.001	-4.219	<0.001	-0.683	0.495	-0.18	0.857
T4	-3.39	0.001	-2.524	0.012	-4.054	<0.001	-0.036	0.972	-2.48	0.013
T5	-2.945	0.003	-1.64	0.101	-1.11	0.267	-1.561	0.119	-1.995	0.046
T6	-1.361	0.173	-4.296	<0.001	-3.005	0.003	-3.258	0.001	-3.386	0.001
Fz	-1.566	0.117	-1.467	0.142	-1.878	0.06	-1.898	0.058	-2.701	0.007
Cz	-2.945	0.003	-2.569	0.01	-2.326	0.02	-2.212	0.027	-0.911	0.362
Pz	-4.002	<0.001	-0.973	0.331	-0.36	0.719	-0.047	0.962	-0.344	0.731

Table 23 The comparisons of CC between before and after hormonal therapy in the IESS group during the sleep ictal phase

Node	delta		theta		alpha		beta		gamma	
	Z	*P*	*Z*	*P*	*Z*	*P*	*Z*	*P*	*Z*	*P*
Fp1	-2.139	0.032	-0.425	0.671	-1.284	0.199	-1.027	0.304	-2.585	0.01
Fp2	-3.894	<0.001	-2.84	0.005	-0.526	0.599	-2.429	0.015	-0.281	0.778
F3	-4.174	<0.001	-0.765	0.444	-1.131	0.258	-3.847	<0.001	-1.888	0.059
F4	-3.465	0.001	-0.533	0.594	-2.592	0.01	-2.347	0.019	-5.328	<0.001
C3	-2.339	0.019	-2.106	0.035	-1.311	0.19	-1.606	0.108	-2.129	0.033
C4	-2.815	0.005	-3.598	<0.001	-0.569	0.569	-1.6	0.11	-1.877	0.061
P3	-2.804	0.005	-0.76	0.447	-2.151	0.031	-1.899	0.058	-1.752	0.08
P4	-2.163	0.031	-2.015	0.044	-2.953	0.003	-1.567	0.117	-1.066	0.287
O1	-2.813	0.005	-1.613	0.107	-1.211	0.226	-3.047	0.002	-3.055	0.002
O2	-3.027	0.002	-0.704	0.481	-2.839	0.005	-0.529	0.597	-2.974	0.003
F7	-4.586	<0.001	-2.336	0.019	-1.472	0.141	-1.171	0.242	-1.623	0.105
F8	-2.335	0.02	-1.306	0.192	-1.852	0.064	-0.053	0.958	-4.622	<0.001
T3	-3.808	<0.001	-1.535	0.125	-2.729	0.006	-3.96	<0.001	-0.292	0.77
T4	-2.079	0.038	-3.821	<0.001	-0.228	0.82	-2.055	0.04	-3.332	0.001
T5	-3.454	0.001	-1.739	0.082	-0.48	0.632	-0.429	0.668	-0.841	0.4
T6	-3.487	<0.001	-2.377	0.017	-2.074	0.038	-0.216	0.829	-1.395	0.163
Fz	-2.213	0.027	-0.048	0.961	-0.85	0.395	-0.387	0.699	-1.126	0.26
Cz	-0.831	0.406	-1.773	0.076	-0.344	0.731	-0.313	0.754	-1.039	0.299
Pz	-2.578	0.01	-0.313	0.755	-1.844	0.065	-0.216	0.829	-2.194	0.028

Table 24 The comparisons of BC between before and after hormonal therapy in the IESS group during the sleep ictal phase

Node	delta		theta		alpha		beta		gamma	
	Z	*P*	*Z*	*P*	*Z*	*P*	*Z*	*P*	*Z*	*P*
Fp1	-1.665	0.096	-1.529	0.126	-0.221	0.825	-2.059	0.039	-4.312	<0.001

Fp2	-5.483	<0.001	-3.02	0.003	-0.95	0.342	-1.244	0.214	-0.371	0.711
F3	-1.788	0.074	-0.979	0.327	-0.439	0.66	-1.821	0.069	-1.388	0.165
F4	-5.125	<0.001	-0.359	0.719	-1.733	0.083	-0.845	0.398	-2.188	0.029
C3	-3.598	<0.001	-0.408	0.683	-0.884	0.377	-6.535	<0.001	-1.49	0.136
C4	-2.127	0.033	-0.285	0.775	-0.124	0.901	-2.997	0.003	-1.315	0.188
P3	-0.949	0.343	-1.008	0.313	-0.712	0.477	-0.392	0.695	-0.982	0.326
P4	-4.398	<0.001	-0.552	0.581	-2.161	0.031	-1.053	0.293	-4.328	<0.001
O1	-2.68	0.007	-1.769	0.077	-1.595	0.111	-2.536	0.011	-1.59	0.112
O2	-2.839	0.005	-0.203	0.839	-2.4	0.016	-0.894	0.371	-2.365	0.018
F7	-2.213	0.027	-0.323	0.747	-0.373	0.709	-1.543	0.123	-0.948	0.343
F8	-0.037	0.97	-2.795	0.005	-5.814	<0.001	-0.012	0.991	-3.917	<0.001
T3	-1.428	0.153	-5.902	<0.001	-1.969	0.049	-0.716	0.474	-0.26	0.795
T4	-2.443	0.015	-0.358	0.72	-4.147	<0.001	-2.889	0.004	-3.109	0.002
T5	-0.956	0.339	-2.284	0.022	-0.936	0.349	-0.702	0.483	-0.048	0.962
T6	-4.221	<0.001	-5.305	<0.001	-3.165	0.002	-1.681	0.093	-2.093	0.036
Fz	-2.301	0.021	-1.703	0.088	-2.296	0.022	-1.185	0.236	-7.424	<0.001
Cz	-0.606	0.545	-1.094	0.274	-2.152	0.031	-4.48	<0.001	-1.265	0.206
Pz	-1.724	0.085	-1.449	0.147	-1.04	0.298	-0.499	0.618	-2.976	0.003
